# Ephrin B1 Regulates Inflammatory Pathways in Retinal Müller Cells

**DOI:** 10.33696/diabetes.6.056

**Published:** 2024

**Authors:** Li Liu, Youde Jiang, Mohamed Al-Shabrawey, Jena J. Steinle

**Affiliations:** 1 Department of Ophthalmology, Visual and Anatomical Sciences, Wayne State University School of Medicine, Detroit, MI 48201, USA; 2 Eye Research Center and Institute, Oakland University William Beaumont School of Medicine (OUWB-SOM), Oakland University, Oakland, MI 48309, USA; 3 Department of Foundational Medical Studies, OUWB-SOM, Oakland university, USA

**Keywords:** Ephrin B1, Inflammation, Diabetes, Müller cells, Retina, Mice, NLRP3

## Abstract

The role of inflammation has been accepted as a factor in the complications of diabetic retinopathy. Discovery of the upstream regulation of these inflammatory factors has remained a challenge. In this study, we explored the actions of ephrin B1 in retinal Müller cells and their actions on inflammatory proteins. We used diabetic human and mouse samples, as well as Müller cells in culture to measure ephrin B1 in Müller cells. We then generated Müller cell specific ephrin B1 knockout mice. We measure levels of key inflammatory proteins, including high mobility group box 1 (HMGB1) and NOD-like receptor protein 3 (NLRP3) pathway proteins in retinal lysates from the ephrin B1 floxed and ephrin B1 Müller cell specific knockout mice. Data show that ephrin B1 is significantly increased in the retina of diabetic humans and mice, as well as in Müller cells grown in high glucose. Elimination of ephrin B1 in mouse Müller cells led to a significant decline in all inflammatory proteins studied. In conclusion, a reduction in ephrin B1 in the diabetic retina may offer a new therapeutic modality for diabetic retinopathy.

## Introduction

For the last several decades, the role of inflammation has been appreciated in the retina [1,2]. This inflammation has been linked to innate immune pathways, toll-like receptor 4 (TLR4) [3], danger pathways through high mobility group box 1 (HMGB1) [4], or the NLR family pyrin domain containing 3 (NLRP3) pathway [5,6]. While it is clear these pathways are all active in the retina, the upstream regulation of this inflammation remains less clear.

The goal of this study was to explore whether ephrin B1 could regulate inflammatory pathways in retinal Müller cells. Ephrin B1 typically binds the Eph B1 receptor and can participate in bidirectional signaling with the Eph B1 receptor [7,8]. In this way, both Eph receptors and ephrin molecules can act as a ligand and a receptor to initiate cellular actions [9]. Eph/ephrins play a key role in embryogenesis through their actions to organize tissues [7,10]. They also regulate angiogenesis, vasculogenesis, and axon guidance [10]. Our goal was to examine ephrin B1, so we choose to focus these studies on retinal Müller cells, as ephrin B1 was localized on Müller cells in glaucoma studies [11].

Müller cells have been shown reported to regulate inflammatory pathways in the retina [12]. Ephrin B1 is also associated with inflammation in other targets. Studies in mice show that injection of ephrin B1-Fc lead to hyperalgesia and is involved in pain sensitization [13]. Further studies on ephrin B1 in pain showed that ephrin B1 regulated mitogen activated protein kinase (MAPK) to mediate its actions [14]. Work in a rheumatoid arthritis model showed that ephrin B1 increased tumor necrosis factor alpha (TNFα) and interleukin-6 (IL-6) actions in lymphocytes [15]. Focusing on the eye, studies in a mouse glaucoma model showed that ephrin B1 led to significant increase in TNFα actions in Müller cells [16]. In contrast to Müller cells actions, ephrin B1 reduced TNFα actions in adipose cells [17].

Based on the literature, we hypothesized that ephrin B1 will increase inflammatory mediators in retinal Müller cells. We will explore TNFα, as well as HMGB1 and NLRP3 pathways.

## Materials and Methods

### Human samples

Dr. Mohamed Al-Shabrawey provided retinal samples from 7 control patients and 7 patients with diabetic retinopathy (both type 1 and type 2). This study was performed in line with the principles of the Declaration of Helsinki. Approval was granted by the Ethics Committee of Oakland University (5/9/23; *IRB-FY2023–292*). These samples and information were evaluated by Oakland University and deemed not human research as part of processed on May 9, 2023 for the study entitled “Molecular and cellular mechanisms of diabetic retinopathy.” All diabetic patients had disease for 10+ years. Since these samples are deidentified and collected postmortem, they are not considered human research. These samples were processed for protein detection by Western blot.

### Diabetic mice

Eight-week-old male C57BL/6 mice were purchased from Jackson Laboratories. Diabetes was induced by 5 days of streptozotocin injections (60mg/kg, I.P) [18]. Glucose levels greater than 250 mg/dl were defined as diabetic. At six months of diabetes, 8 control and 8 diabetic mice were sacrificed for analyses as we have done in the past [3]. No animals died before reaching the 6-month time point. The body weight and blood glucose of the mice is shown in Table 1. Mice were allowed free access to water and food and kept at a constant temperature for all experiments. Mice were checked weekly for health. At the time of euthanasia, mice were euthanized by CO_2_ overdose and cervical dislocation. All mouse experiments were approved by the Wayne State University IACUC committee and adhere to the rules provided by the ARVO animal care groups.

### Genotyping of Ephrin B1 mice

Genomic DNA was extracted from ear punch samples from 3-week-old mice. Ear punches were digested with one step tail DNA extraction buffer (100 mM Tris, 5 mM EDTA, 200 mM NaCL, 1% Triton) plus proteinase K (10 mg/ml) at 55°C overnight, followed by enzyme heat-inactivation at 85°C for 45 min. Primer pairs used to screen the Ephrin conditional knock out mice were as follows: Ephrin mutant forward: 5’->3’: GGC CTT TGA AGG AAT GTG AA, reverse 5’->3’: TTG TCC TAA TGG GGC ATT TC. Pdgfra-cre 5’->3’ forward: GCG GTC TGG CAG TAA AAA CTA TC and reverse: GTG AAA CAG CAT TGC TGT CAC TT. The PCR reaction was done using KAPA2G HotStar Genotyping PCR Mix (KK5621, KAPA Biosystems). PCR reaction was performed with following temperatures and times: denature: 95°C 3 min, 35 cycles at 95°C, 15 sec, 60°C 15 sec, 72°C sec/kb, and a final extension at 72°C 1 min.

### Immunostaining rMC-1 cell culture

rMC-1 (rat Müller cells) cells were provided by Vijay Sarthy (Northwestern University). Cells were grown on chamber slides (Thermo Scientific Nunc Lab-Tek^™^) in DMEM medium (normal (5 mM) glucose) supplemented with 10% fetal bovine serum and antibiotics. After 5 days, the media was removed, and cells were placed into 4% paraformaldehyde for 1 hour. After several washes in PBS, slides were blocked with 5% bovine serum albumin (BSA) for 1h at room temperature to eliminate nonspecific staining, followed by incubation with rabbit anti ephrin B1 (1:400, Invitrogen) at 4°C overnight. After rinsing in 0.1% Triton/PBS, slides were incubated with secondary antibody donkey anti rabbit Alexa 555 (1:500, Life Technologies) for 2h at room temperature. Slides were then rinsed in PBS, mounted with FluorSave Reagent (Calbiochem), and examined on a Leica Confocal microscope.

### Mice tissue

Two-month-old male and female ephrin B1 floxed mice and ephrin B1 PDGFAR- cre KO mice were euthanized by CO_2_, followed by cervical dislocation. After confirmation of death, eyes were removed and placed into in 4% paraformaldehyde in PBS for 6 hours. Whole globes were transferred into 0.1M PBS with 30% sucrose overnight for cryoprotection, then 10 μm cryosections were collected and stored in −20°C for further analysis. Slides were rinsed in PBS and placed into 5% normal goat serum for 2h at room temperature to block nonspecific staining, followed by incubation with rabbit anti-ephrin B1 (1:400, Invitrogen) and mouse anti-glutamine synthetase (1:500, Abcam) overnight at 4°C. After rising in 0.3% Triton/PBS, slides were incubated with secondary antibody goat anti-rabbit conjugated to Alexa Fluor 555 (1:500, Life Technologies) and goat anti mouse conjugated to Alexa Fluor 488 (1:500, Life Technologies) overnight at 4°C. Slides were then rinsed in PBS, counter staining with DAPI, mounted with FluorSave Reagent (Calbiochem), and examined on a Leica Confocal microscope.

### Western blotting

Cell or retinal lysates were collected into lysis buffer containing protease and phosphatase inhibitors. Equal amounts of protein were separated onto tris-glycine gels (Invitrogen, Carlsbad, CA), and blotted onto a nitrocellulose membrane. After blocking with TBST (10mM Tris-HCl buffer, pH 8.0, 150 mM NaCl, 0.1% Tween 20) and 5% (w/v) BSA, the membranes were treated with ephrin B1 (1:500, Proteintech), NLRP3 (1:1200, Abcam), TNFα (1:200, Abcam), IL-1β (1:200, Abcam), cleaved caspase 1 (1:200, Abcam), Nek7 (1:1000, Abcam), HMGB1 (1:500, Abcam) and beta actin (Santa Cruz Biotechnology, Santa Cruz, CA) primary antibodies followed by incubation with secondary antibodies labeled with horseradish peroxidase. Antigen-antibody complexes were detected by chemilluminescence reagent kit (Thermo Scientific, Pittsburgh, PA) and data was acquired using an Azure C500 (Azure Biosystems, Dublin, CA). Western blot data were assessed using Image Studio Lite software.

### Statistics

Prism software 9.0 (GraphPad, La Jolla, CA) was used for statistical analyses. A one-way ANOVA with Tukey’s post-hoc test or a T-test were used for analyses with *P*<0.05 taken as significant. A representative blot is provided for Western blot data.

## Results

### Ephrin B1 levels increased in retina from diabetic patients and diabetic mice

**Figure 1.**
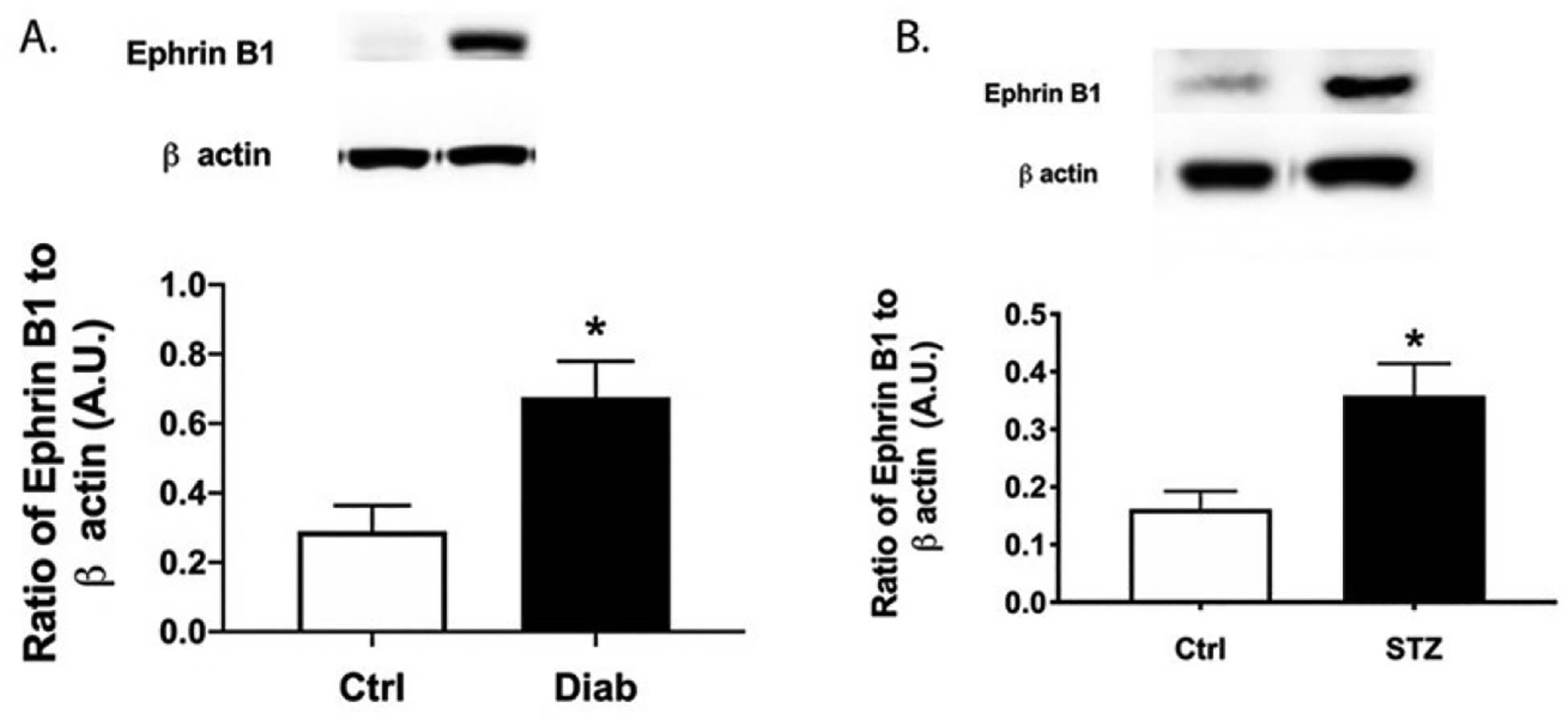
Ephrin B1 is increased in diabetic human and mouse samples. Western blotting was done on human retinal samples **(A)** and control and diabetic mice **(B).** N=7 for humans and N=8 for mice. Data is mean ± SEM. *P<0.05 vs. Ctrl.

Using protein samples collected from patients with type 1 and type 2 diabetes, we found significantly increased levels of ephrin B1 in the retina (Figure 1A). Similarly, ephrin B1 was significantly increased in the retina from mice treated with streptozotocin (Figure 1B).

### Ephrin B1 protein levels are increased in Müller cells grown in high glucose

**Figure 2.**
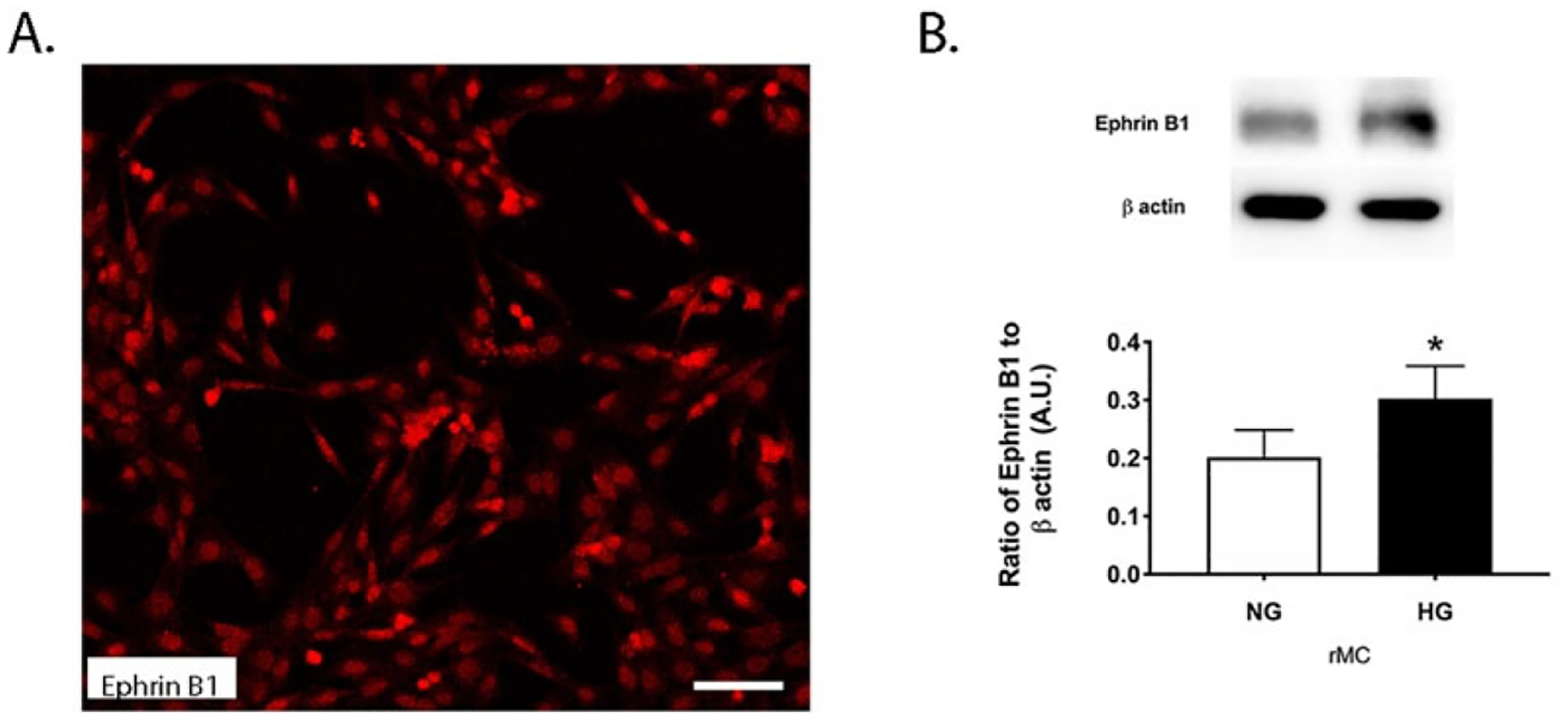
Ephrin B1 is localized in retinal Müller cells. rMC-1 cells were cultured in normal (5 mM) and high (25 mM) glucose. **Panel A** shows localization of ephrin B1 in rMC1 cells, and **Panel B** shows Western blot data from rMC-1 cells. N=5. Data is mean ± SEM. P<0.05 vs. NG for panel B. Scale bar is 50 um.

To support our findings from the mice and patients, we also grew rMC-1 cells in normal and high glucose. Some cells grown in normal glucose were plated onto chamber slides for immunostaining for ephrin B1. Figure 2A shows ephrin B1 localized in Müller cells. Other cells were collected into lysis buffer for Western blotting. Figure 2B shows that protein levels of ephrin B1 were significantly increased in retinal Müller cells grown in high glucose.

### Generation of the Müller cell specific ephrin B1 knockout mouse line

**Figure 3.**
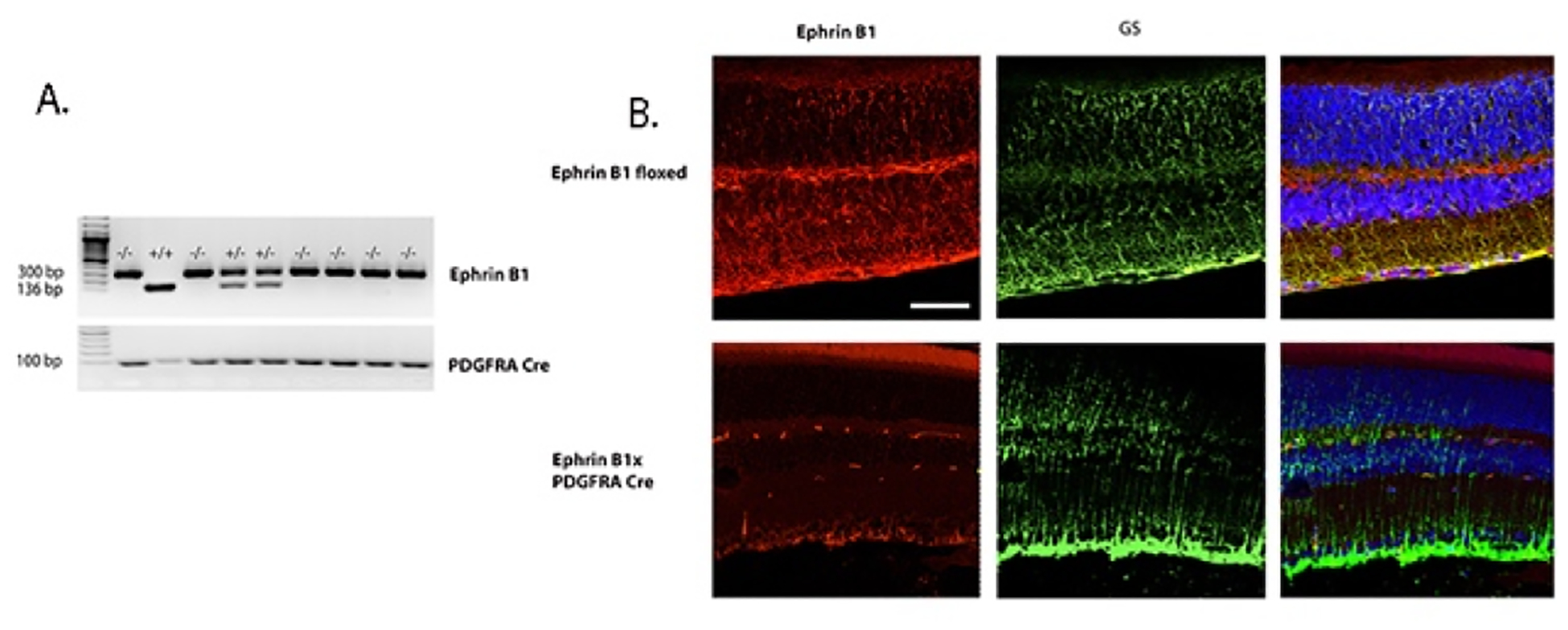
Generation of a Müller cell specific ephrin B1 knockout mouse line. **Panel A** shows genotyping from ephrin B1 floxed and ephrin B1 x PDGFRα Cre mice. **Panel B** shows immunostaining from each set of mice for ephrin B1 (red) and glutamine synthase (green). The far right panel is a merging of the other two panels. Scale bar is 50 um.

To better understand ephrin B1 actions in retinal Müller cells, we crossed ephrin B1 floxed mice with PDGDRα Cre mice to eliminate ephrin B1 in retinal Müller cells. Figure 3A shows the genotyping for some of these mice. To support our genotyping data, we also performed immunostaining for ephrin B1 (red) and glutamine synthase (GS, green) to demarcate ephrin B1 in retinal Müller cells on sectioned retina from the ephrin B1 floxed and ephrin B1 x PDGFRα Cre mice. Figure 3B shows that there is more yellow staining in the ephrin B1 floxed vs. the knockout mice.

### Ephrin B1 regulates HMGB1 and TNFα levels

**Figure 4.**
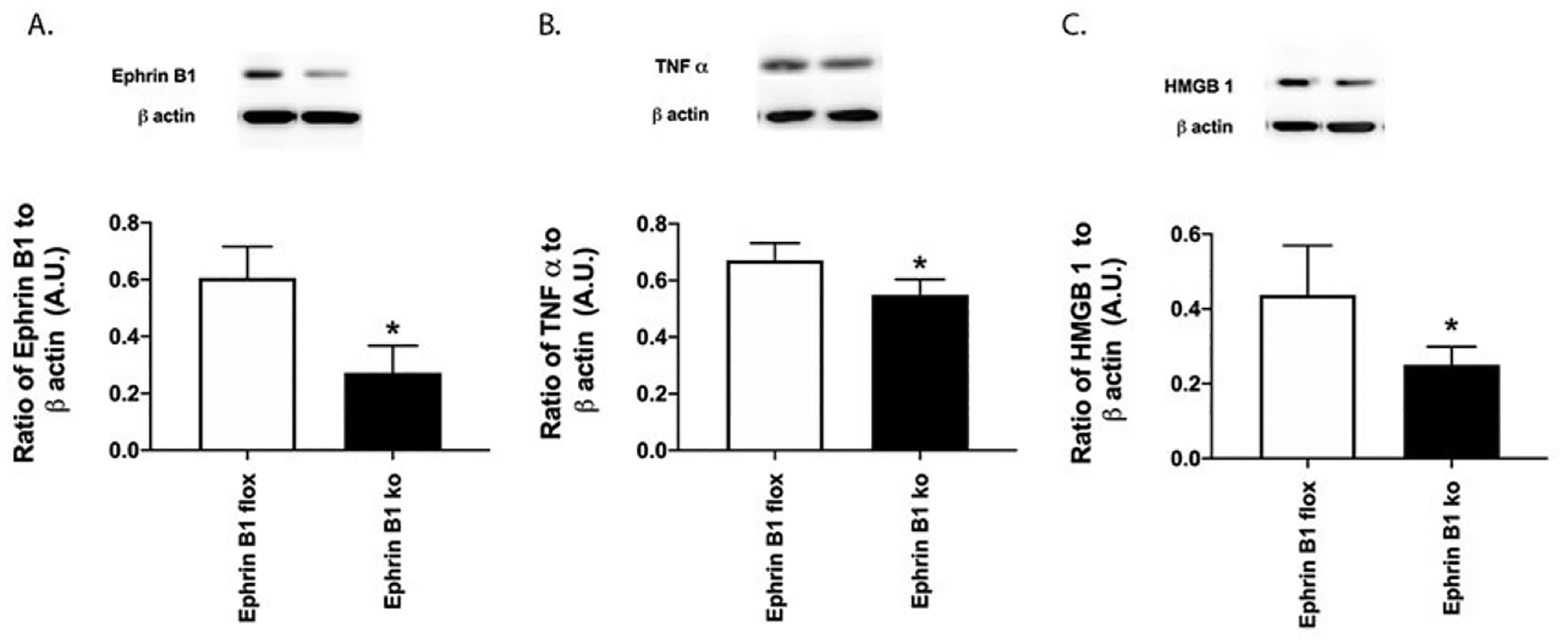
Ephrin B1 regulates inflammatory protein in mouse retina. Western blotting was done in whole retinal lysates from ephrin B1 floxed and ephrin B1 floxed x PDGFRα Cre (Ephrin B1 KO) for ephrin B1 **(A)**, TNFα **(B)**, and HMGB1 **(C)**. N=8 for each group. Data are mean ± SEM. *P<0.05 vs. ephrin B1 floxed.

Since ephrin B1 regulates inflammatory mediators in other systems, we explored this in the knockout mice. Figure 4A is a control to show reduced ephrin B1 protein levels in the Müller cell specific knockout mice. Figure 4B and 4C show a significant reduction in TNFα and HMGB1 levels in the retina from mice where ephrin B1 is eliminated in Müller cells.

### Ephrin B1 regulates the NLRP3 signaling pathway

**Figure 5.**
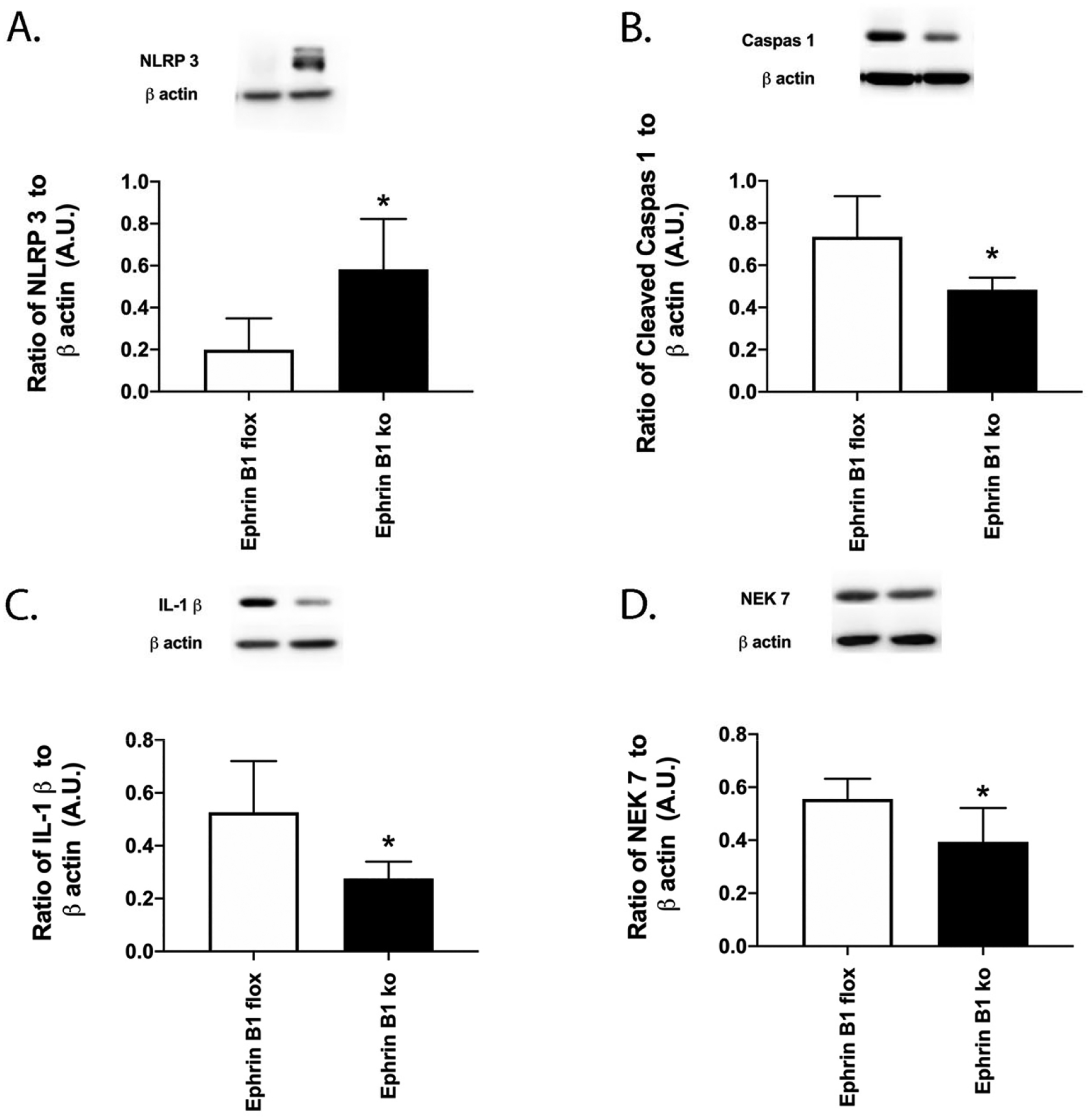
Ephrin B1 regulates NLRP3 in mouse retinal Müller cells. Western blotting was done in whole retinal lysates from ephrin B1 floxed and ephrin B1 floxed x PDGFRα Cre (Ephrin B1 KO) for NLRP3 **(A)**, cleaved caspase 1 **(B)**, IL-1β **(C)** and Nek7 **(D)**. N=8 for each group. Data are mean ± SEM. *P<0.05 vs. ephrin B1 floxed.

In addition to HMGB1 and TNFα, we also measured proteins in the NLRP3 pathway in the mice. Figure 5 shows that loss of ephrin B1 in retinal Müller cells significantly reduced NLRP3 (A), cleaved caspase 1 (B), IL-1β (C) and Nek7 (D). These data, combined with Figure 4, suggest that reduction of ephrin B1 actions may provide a viable treatment option for diabetic retinal disease.

## Discussion

Literature supports a role for inflammation in the diabetic retina [19]. We have shown a strong role for upstream regulation of inflammatory pathways in retinal endothelial cells (REC) [3,20,21]. Our goal in these studies was to expand our work to explore ephrin B1 as a key regulator of inflammatory pathways. Ephrin B1 has been localized to Müller cells in other animal models, so we targeted ephrin B1 in mouse retinal Müller cells. Little work has been done to investigate ephrin B1 in the diabetic retina. Our data show significantly increased ephrin B1 protein levels in the diabetic human and diabetic mouse retina. High glucose significantly increased ephrin B1 levels in rMC-1 Müller cells in culture.

Ephrin B1 has been linked to inflammation in other models. Literature in pain models showed that ephrin B1 led to increased hyperalgesia [13]. Others have also shown a role for ephrin B1 and TNFα in rheumatoid arthritis models [15]. Little has been done to investigate ephrin B1 actions on HMGB1 or the NLRP3 pathway. Both inflammatory pathways are linked to diabetic retinopathy [5, 22]. In this study, we show for the first time a role for ephrin B1 regulation of both HMGB1 and NLRP3 in retinal Müller cells. Future work can explore the mechanisms behind this regulation.

While we can study protein levels of key proteins in human samples or human fluids, it is difficult to manipulate the findings to explore potential mechanisms of disease. The goal of this study was to develop a mouse model of a human-type disease (rodents do not develop proliferative retinopathy), with a focus on Ephrin B1. We can use this model to better explore how the loss of Ephrin B1, specifically in Müller cells, can cause some retinal damage that is also reported in human diabetic patients. It does not replace the need for studies in humans, but it does allow for some work on potential mechanisms of disease.

While many of the findings in this study are novel, there are limitations to our studies. The PDGFRα Cre mice could target other cell types; however, we do show loss of staining in Müller cells in the mice. Additionally, we did not explore ephrin B1 actions in other cell types. Future work can explore ephrin B1 in REC or other relevant cell types in diabetic retinopathy.

In conclusion, these studies show that ephrin B1 is significantly increased in the diabetic retina and in retinal Müller cells. Loss of ephrin B1 only in retinal Müller cells significantly reduced inflammatory markers, including TNFα, HMGB1, and NLRP3. These studies lay the groundwork for future work on the mechanisms by which ephrin B1 reduces inflammation in the diabetic retina.

## Figures and Tables

**Table 1.** Data are mean ± St. Dev.

C57BL/6				
	Ctrl		STZ	
	BW (g)	BG	BW (g)	BG
8 weeks of age	25.6 ± 2.2	113 ± 12	25.2 ± 2.0	110 ± 8.1
6 months of age	35 ± 2*	119 ± 8	26 ± 1.4	397 ± 119^#^

* p<0.05 vs. 8 weeks in control

# p<0.05 vs. 8 weeks in STZ.
